# Fungal Communities Including Plant Pathogens in Near Surface Air Are Similar across Northwestern Europe

**DOI:** 10.3389/fmicb.2017.01729

**Published:** 2017-09-08

**Authors:** Mogens Nicolaisen, Jonathan S. West, Rumakanta Sapkota, Gail G. M. Canning, Cor Schoen, Annemarie F. Justesen

**Affiliations:** ^1^Department of Agroecology, Aarhus University Slagelse, Denmark; ^2^Biointeractions and Crop Protection Department, Rothamsted Research (BBSRC) Harpenden, United Kingdom; ^3^Wageningen University & Research Wageningen, Netherlands

**Keywords:** airborne, plant pathogen, metabarcoding, air sampling, urban

## Abstract

Information on the diversity of fungal spores in air is limited, and also the content of airborne spores of fungal plant pathogens is understudied. In the present study, a total of 152 air samples were taken from rooftops at urban settings in Slagelse, DK, Wageningen NL, and Rothamsted, UK together with 41 samples from above oilseed rape fields in Rothamsted. Samples were taken during 10-day periods in spring and autumn, each sample representing 1 day of sampling. The fungal content of samples was analyzed by metabarcoding of the fungal internal transcribed sequence 1 (ITS1) and by qPCR for specific fungi. The metabarcoding results demonstrated that season had significant effects on airborne fungal communities. In contrast, location did not have strong effects on the communities, even though locations were separated by up to 900 km. Also, a number of plant pathogens had strikingly similar patterns of abundance at the three locations. Rooftop samples were more diverse than samples taken above fields, probably reflecting greater mixing of air from a range of microenvironments for the rooftop sites. Pathogens that were known to be present in the crop were also found in air samples taken above the field. This paper is one of the first detailed studies of fungal composition in air with the focus on plant pathogens and shows that it is possible to detect a range of pathogens in rooftop air samplers using metabarcoding.

## Introduction

Aerial dispersal of spores over short or long distances affects the epidemiology of many fungal plant pathogens, and long-distance dispersal is an important strategy for a number of pathogens which may lead to invasion into new areas or spread of aggressive races of pathogens on the global scale (Brown and Hovmoller, 2002). The amount and timing of air dispersal of spores of individual fungal plant pathogens has been studied with the aim to model disease pressures and in the end to be able to predict disease risks in decision support systems. However, the mechanisms of how air mass is mixed, the origins and release of spores, and finally deposition of spores on plant leaves are not very well-studied (Schmale and Ross, 2015). Previously, airborne spores were identified by microscopy which is a lengthy process that can only identify relatively large spores with visual characteristics. The introduction of immunological and DNA based methods have largely facilitated the detection and quantification of specific fungal species in air samples. Kennedy et al. (2000) used a microtiter immunospore trapping device (MTIST device) to monitor airborne inoculum of *Mycosphaerella brassicicola* and *Botrytis cinerea* and found a relationship between airborne inoculum densities and severity of disease on plants in the field. *Ramularia beticola* in air samples could be detected by qPCR 14 to 16 days prior to first visible symptoms on sugar beet and the assay was suggested as part of an early warning system (Wieczorek et al., 2013). Carisse et al. (2009b) developed a sensitive TaqMan based qPCR assay for quantification of *Botrytis squamosa* spores in air and demonstrated the potential as a disease risk predictor in onion. In another study, periods of high risk of grape powdery mildew could be established using measurements of conidia in air (Carisse et al., 2009a). Despite that temporal and spatial dispersal of airborne spores of a few plant pathogens has been studied in detail (Brown and Hovmoller, 2002; Rogers et al., 2009; Klosterman et al., 2014), fungal diversity in air and the impact on plant disease has been disproportionally understudied, mostly due to previously insufficient methods that were only able to analyze a small fraction of the total fungal diversity.

Novel sequencing technologies have enabled insights into microbial communities with an unprecedented resolution (Caporaso et al., 2011). These technologies coupled with air sampling devices could provide novel insights into the diversity of microorganisms in air, an environment in which bacteria, archaea, fungi, and plant spores and pollen are abundant with up to millions of cells/m^3^ (Lighthart, 2000). Previous studies of airborne microbial diversity using next generation sequencing technology have focused on allergenic or human pathogenic organisms whereas plant pathogens have received comparatively little attention (Adams et al., 2013). Using metabarcoding, a very high diversity of airborne fungi was found in the air: Fröhlich-Nowoisky et al. (2009) estimated, by cloning and sequencing, more than 1000 airborne fungal species at a German site and found remarkable seasonal variation for different fungal genera such as *Alternaria*, *Cladosporium* and *Blumeria*, probably reflecting different life-styles, phenological differences and substrate preferences of the different genera. Similarly, pyrosequencing of air samples from an urban setting resulted in the identification of more than 1000 fungal OTUs (operational taxonomic units) of which most showed seasonal variation (Yamamoto et al., 2012).

In the present study, we sampled spores from air during 10-day periods in spring and autumn from three locations in Northwestern Europe (Denmark, England, and The Netherlands), from rooftops remote of agricultural fields. Those three locations have approximately similar climates. The spore traps were placed on top of tall buildings in urban surroundings in order to sample well-mixed air and to avoid sampling bias from single close-by fields. The aim was to obtain air samples which were representative of the fungal species composition in air at a regional scale (West and Kimber, 2015). For reference, a spore trap was operated at ground level in experimental oilseed rape fields at Rothamsted. Autumn and early spring were chosen as sampling periods to allow for detection of plant pathogens in periods where we assume that spores would be important in initiating new infections in fields with either autumn or spring-sown crops. The content of fungal spores in the collected samples was analyzed using metabarcoding of the fungal ITS1 region and by qPCR for selected pathogens. By doing this, we wanted to answer the following questions: what is the level of diversity of fungi in air and what are the proportions of plant pathogenic fungi, and finally, what are the drivers of variation in those communities?

## Materials and Methods

### Study Sites and Sampling

The study sites were located in Slagelse, Denmark (GPS coordinates 55.405343° N, 11.355863° E) (approximate height above ground level: 15 m); Rothamsted, UK (51.809568° N, 0.356322° W) (approximate height: 10 m); and Wageningen, The Netherlands (51.986500° N, 5.663025° E) (approximate height: 15 m), respectively. Slagelse is a city with a population of 30,000 and is located in a rural area with cereals and rape as the main crops. Wageningen has a population of 40,000 and is surrounded by arable land and woodland. Rothamsted is located on the edge of a small town (30,000 inhabitants) surrounded by primarily arable farmland with additional areas of permanent grassland and woodland. Furthermore, sampling at ground level took place in experimental fields at Rothamsted. This was in field ‘Geescroft’ in spring 2012 (1 km SSW of the rooftop site), and field ‘Little Hoos’ in spring 2013 (1.3 km W of the rooftop site), both of which contained oilseed rape crops that had been artificially inoculated with *Sclerotinia sclerotiorum*. The air sampler was raised to the same height as the crop canopy (approximately 1 m).

Sampling was performed using a Burkard 7-day recording volumetric spore trap (Burkard Manufacturing, Co., Rickmansworth, United Kingdom). The air flow was set according to Burkard’s standard settings at 10 L min^-1^ (14.4 m^3^ per 24 h). Melinex^®^ tape used for spore trapping was prepared by coating the tape with a thin film of adhesive to trap spores. The adhesive was made in aseptic conditions and was a mixture of approximately 78% petroleum jelly (Vaseline), 20% paraffin wax (melting point 48°C) and 2% phenol, which was applied as a liquid dissolved in hexane, which was allowed to evaporate to leave a thin film (Lacey and West, 2006). Tapes were replaced each week at the same time, cut into segments (48 mm × 20 mm) representing 24 h from 0 a.m. to 12 p.m. (in the following referred to as 1 day) and stored at 6°C. In 2012, spore sampling was performed in ‘early spring’ (April 20, 2012 to April 30, 2012); ‘late spring’ (May 31, 2012 to June 9, 2012) and ‘autumn’ (November 3, 2012 to November 13, 2012). In 2013, spore sampling was performed in ‘early spring’ (May 3, 2013 to May 12, 2013) and ‘late spring’ (June 7, 2013 to June 16, 2013). Weather data (mean temperature and precipitation) for the respective locations and periods can be found in **Supplementary Table S2**.

### DNA Extraction, qPCR, PCR Amplification, and Metabarcoding

Exposed 7-day tape samples from the Burkard spore traps were handled separately in a clean lab and each cut into daily sections, 48 mm × 20 mm. Each daily section was sub-divided lengthways into two sections 48 mm × 10 mm, each placed in a 2 ml tube and stored at -20°C. One of the two DNA extractions representing a daily section was used for sequencing analysis; the other was stored as a back-up. To each tube 0.5 g of sterile glass beads (425–600 μm diameter; Sigma) were added together with 440 μl of extraction buffer [2x TEN (500 mM NaCl, 400 mM Tris-HCl, 50 mM EDTA, pH 8; 0.95% SDS; 2% polyvinylpyrrolidone; 5 mM 1,10-phenanthroline monohydrate, and just before use 0.1% β-mercaptoethanol)] and tubes were then shaken in a FastPrep machine (Savant FastPrep BIO101 Homogenizer, Thermo Fisher, Waltham, MA, United States) three cycles of 6.0 m s^-1^ for 40 s, with 2 min cooling on ice between cycles. 400 μl 2% SDS (sodium dodecyl sulfate) was added, tubes inverted and incubated at 65°C in a water bath for 30 min. Then, 800 μl of phenol:chloroform (1:1) was added, each tube vortexed briefly and then centrifuged at 15,115 *g* for 10 min in a centrifuge (4°C). The supernatant was stored at -20°C overnight. Tubes were then centrifuged at 15,115 *g* for 30 min at 4°C and the pellet was washed with 200 μl of 70% ethanol and centrifuged at 15,115 *g* for 15 min. The DNA pellet was air-dried and resuspended in 30 μl of water at 65°C for 5 min before being stored at -20°C.

To generate ITS1 amplicons for 454 pyrosequencing, primers ITS1-F (Gardes and Bruns, 1993) and 58A2R (Martin and Rygiewicz, 2005) were used; these primers amplify the fungal ITS1 region but not plant DNA (Xu et al., 2012; Nicolaisen et al., 2014). Ten-nucleotide multiplex identifier (MID) primer tags and PrimerA and PrimerB primers were recommended by Eurofins MWG GmbH (Ebersberg, Germany). Primer structures were: 5′-PrimerA-MID-ITS1F-3′ and 5′-PrimerB-58A2R-3′. Primers were synthesized by Eurofins MWG. PCR reactions contained 1× PCR reaction buffer, 1.5 mM MgCl_2_, 0.2 mM dNTPs, 1 μM each primer, 1 U of Taq DNA recombinant polymerase (Promega Corporation, Madison, WI, United States) and 1 μl (app. 5 ng) of DNA template in a final volume of 25 μl. All amplifications were performed in a GeneAmp PCR System 9700 thermal cycler (Thermo Fisher Scientific) using a DNA denaturation step of 94°C for 5 min, followed by 35 cycles at 94°C for 30 s, 48°C for 30 s, 72°C for 1 min, and a final elongation step at 72°C for 10 min. The amount of amplicons was estimated by visual inspection after gel electrophoresis. Tagged PCR amplicons were pooled in equimolar amounts and resolved in 1.5% agarose gels. The visible smear of PCR products at approximately 280–360 base pairs was excised and purified using QIAquick Gel Extraction Kit (QIAGEN GmbH, Hilden, Germany). These libraries were sequenced by Eurofins MWG on a GS Junior 454 Sequencer using titanium chemistry. Distribution of samples in sequencing runs is shown in **Supplementary Table S1**.

The primers that were used for pyrosequencing were not able to efficiently amplify DNA from the genus *Puccinia* as also observed in previous studies (Sapkota et al., 2015). As *Puccinia* contains important plant pathogens, we quantified the amount of *Puccinia striiformis*, *P. graminis*, and *P. triticina* using qPCR. qPCR was performed using 2 μl extracted DNA in a final volume of 10 μl qPCR reaction with a TaKaRa Premix Ex Taq (Perfect Real Time) master mix (Takara Bio, Otsu, Japan), ROX Reference Dye II, 300 nM of each primer and 100 nM of the probe (**Supplementary Table S4**). All the real-time amplifications were performed in a CFX384 (Bio-Rad, Hercules, CA, United States) using a DNA denaturation step of 95°C for 2 min, followed by 40 cycles at 95°C for 15 s and 60°C for 1 min. The concentration of the samples was automatically scored on the basis of the dilution series of the PCR products generated from the reference material. DNA of reference material of *Leptosphaeria maculans* (CBS 147.24), *Microdochium nivale* (IPO1.21), *S. sclerotiorum* (20090907), *P. graminis* (TR112/12), *P. striiformis* (SE35/11), and *P. triticina* (DK57/00) was generated by PCR amplification as described before and quantified using Quant-iT^TM^ PicoGreen (Invitrogen, Carlsbad, CA, United States) in an Infinite 200 PRO monochromator (Tecan, Männedorf, Switzerland). Fungal gene copy numbers were calculated using estimated genome sizes of the species, and the measured DNA quantities.

### Bioinformatics and Statistical Analysis

Raw sequence files were converted into flowgrams and sequences were analyzed using QIIME v. 1.8 (Caporaso et al., 2010). Flowgrams were subjected to Ampliconnoise to remove reads with mismatching primers and MID sequences, PCR and sequencing errors, and chimeras (Quince et al., 2011). ITS1 sequences were extracted using ITSx extractor version 1.0.6 (Bengtsson-Palme et al., 2013). Reads were then clustered using the pick_open_reference_otus.py script at 97% similarity level in UClust (Edgar, 2010). The UNITE database version 6 for QIIME was used as a reference file for OTU picking and assigning taxonomy (Abarenkov et al., 2010; Kõljalg et al., 2013). For species identification, a representative sequence from each OTU with at least 100 sequences in total was subjected to Basic Local Alignment Search Tool (BLAST) matches at NCBI in cases when the UNITE database was unable to assign OTUs to species level.

All singletons were removed before constructing OTU tables. Diversity analysis was carried out using the core_diversity_analyses.py script in QIIME (Caporaso et al., 2010). Non-phylogenetic diversity estimates using observed species numbers for α diversity and Bray–Curtis for β diversity calculations were carried out. Sub-sampling to a minimum sequencing depth of 478 reads per sample was used for analysis of the entire dataset and samples having fewer reads were removed. Non-phylogenetic diversity was calculated separately for year, season and location. In order to compare fungal community composition in different categories and to partition the variance in different categories, Bray–Curtis distance matrices were subjected to Permutational MANOVA (Anderson, 2001) using the *adonis* function with a permutation number of 999 available in the vegan package of R (Oksanen et al., 2013).

Reads and metadata have been published in NCBI SRA with the accession number SRP114941 and the Bioproject number PRJNA397397.

## Results

### Data Characteristics

In total 152 DNA samples each representing 24 h of air sampling from rooftops at three locations and from different seasonal periods, and 41 samples from above agricultural fields in Rothamsted were subjected to pyrosequencing of the fungal ITS1 region. Sequencing resulted in a total of 533,641 reads after quality filtering and ITS extraction. Each sample contained between 8 and 20,338 reads (2765 ± 3082). Samples with low numbers of reads (<478 reads) were excluded from further analysis resulting in a dataset consisting of 160 samples (136 rooftop samples and 24 field samples). Reads could be assembled into 4390 OTUs (excluding singletons), OTUs representing >100 reads in total are shown in **Supplementary Table S1** with total number of reads, taxonomic assignments, and relative quantities listed for each sample. A species accumulation curve indicated that sequencing and sampling depths did not cover the full diversity in air as the curve did not reach a plateau (**Supplementary Figure S1**).

**FIGURE 1:**
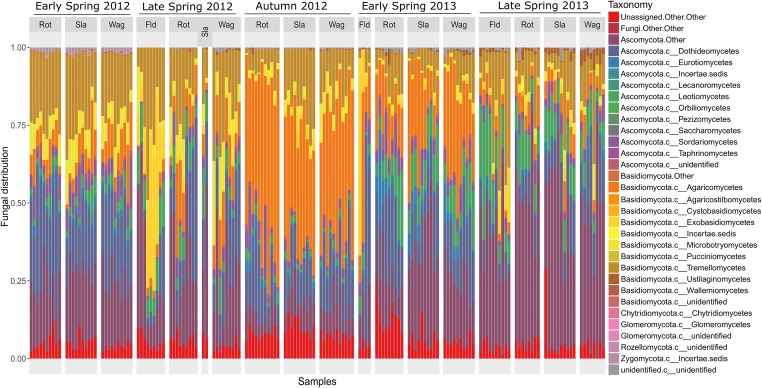
Barchart showing the relative taxonomic distribution of fungal classes in air samples each representing 1 day of sampling at rooftops in Rothamsted, UK, Slagelse, DK and Wageningen, NL, and above oilseed rape fields in Rothamsted during five periods in 2012 and 2013 (early spring 2012, late spring 2012, autumn 2012, early spring 2013, late spring 2013). Rot = Rothamsted, UK; Sla = Slagelse, DK; Wag = Wageningen, NL; Fld = Rothamsted oilseed rape field samples.

**FIGURE 2:**
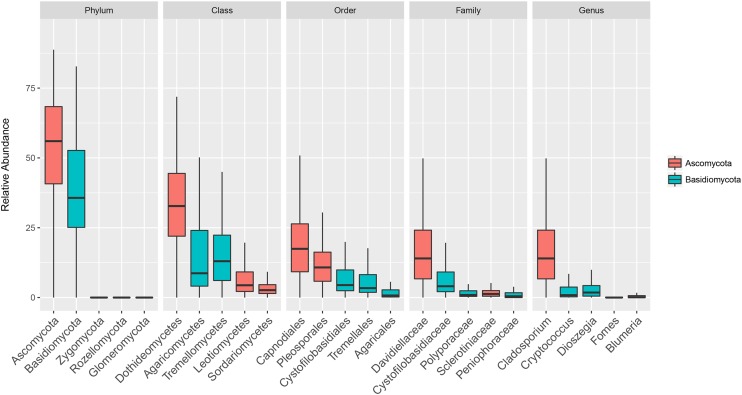
Boxplot showing total relative abundance of reads of the five most abundant taxa at Phylum, Class, Order, Family and Genus levels in 152 rooftop air samples from 2012 and 2013 in Rothamsted, UK, Slagelse, DK, and Wageningen, NL. Upper, middle and lower lines represent first quartiles, medians and third quartiles. The whiskers represent a 1.5 ^∗^ inter-quartile range.

The composition of the communities in each sample is shown at class level in **Figure 1**, and **Figure 2** shows the overall composition of fungal communities at Phylum, Class, Order, Family, and Genus levels. Most of the detected fungi were Ascomycota (53.4%) and Basidiomycota (39.6%). The proportion of Basidiomycota was highest in autumn 2012, which was primarily caused by a high abundance of Agaricomycetes (gilled mushrooms) in the samples (**Figure 1**). The dominant classes in Ascomycota were Dothideomycetes (16.0%), Leotiomycetes (6.8%), Sordariomycetes (3.7%) and Eurotiomycetes (1.8%), and in Basidiomycota the classes Agaricomycetes (17.3%), Exobasidiomycetes (2.9%), Microbotryomycetes (4.0%) and Tremellomycetes (14.5%) were the most dominant (**Figure 2**). The proportion of classes within sampling periods was generally constant, whereas variation was observed between seasons (**Figure 1**). One OTU, SH216250.07FU_EF679363_refs consisted of 110431 reads (20.7% of all reads) (**Supplementary Table S1**), this OTU was classified by QIIME only to phylum level as belonging to Ascomycota. However, by BLAST matches of representative reads in NCBI we could assign this OTU to *Cladosporium.*

### α-Diversity of Communities

**FIGURE 3:**
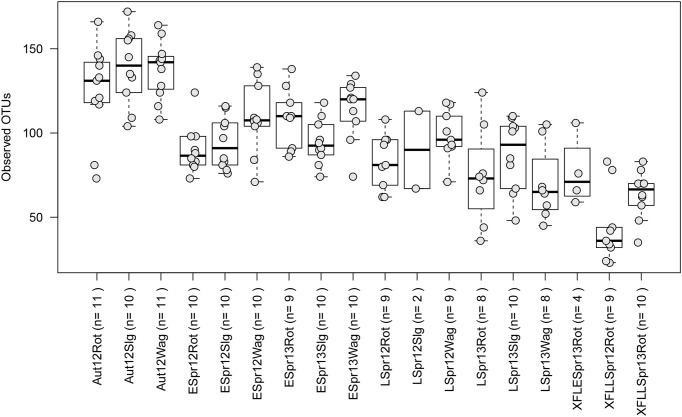
Boxplots showing the number of observed operational taxonomic units (OTUs) for the different sampling periods. Each point represents samples, and upper, middle and lower lines represent first quartiles, medians and third quartiles. The whiskers represent a 1.5 ^∗^ inter-quartile range. Aut = autumn; Espr = early spring; LSpr = late spring; 12 = 2012; 13 = 2013; Rot = Rothamsted; Slg = Slagelse; Wag = Wageningen; X = Rothamsted oilseed rape field samples. n = number of samples.

Fungal communities consisted of ∼25 to 175 observed OTUs in each sample (**Figure 3**). Location did not have a significant effect on richness per sample across all categories (Rothamsted, mean = 95: Slagelse, mean = 103; Wageningen, mean = 107; *p* > 0.05, two-sided Student’s two-sample *t*-test). In contrast, season had a significant effect on richness per sample (Early spring, mean = 100; Late spring, mean = 83; Autumn, mean = 131; *p* < 0.01, all combinations). Generally, samples taken from above fields had lower richness (44.2–67.3) than samples taken from rooftops (94.7–107.3) (**Supplementary Table S3**). The effect of season at individual locations was tested using the Kruskal–Wallis rank sum test after separating data based on location. Except the field samples (*p* = 0.1), fungal richness significantly differed across seasons in Rothamsted (*p* = 0.002), Wageningen (*p* < 0.001), and Slagelse (*p* < 0.001).

### β-Diversity of Communities

**FIGURE 4:**
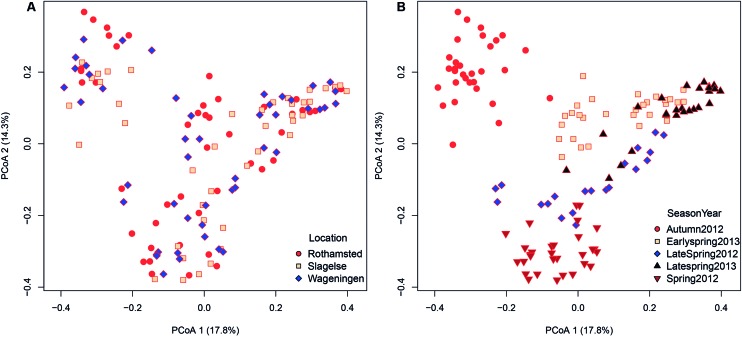
Bray–Curtis matrices visualized using principal coordinates analysis (PCoA) (axes 1, 2, and 3) showing **(A)** distribution of samples according to location (Rothamsted, Slagelse, and Wageningen) in the dataset from rooftop samples, **(B)** distribution of samples according to season (early spring 2012, late spring 2012, autumn 2012, early spring 2013, late spring 2013) in the dataset from rooftop samples.

Since only a few field samples were available these were excluded from the β diversity analysis. Location explained 8% of the variation in the fungal community structures (*adonis R*^2^ = 0.080, *p* < 0.001). A principal coordinates analysis (PCoA) did not show any distinct clustering with regard to the three different rooftop locations in Rothamsted, Slagelse, and Wageningen (**Figure 4A**). However, although location only explained 8% of the variation between communities, some taxa were highly specific for some locations. For example, the genus *Fomes* (a bracket fungus) was found in almost all samples from Slagelse and Wageningen at a relatively high abundance, but was almost absent in samples from Rothamsted (**Supplementary Table S1**).

In contrast, season and year were more important in shaping community structures and explained 24.6% (*adonis R*^2^ = 0.246, *p* < 0.001) of the variation in the dataset. As shown in the PCoA plot (**Figure 4B**), fungal communities clustered according to both year and also season of the year. Samples from autumn were most distinctly separated, but also separation between early and late spring could be observed.

### Plant Pathogens in Air Samples

The following taxa, which are known to contain mainly plant pathogens, were identified (species identifications by BLAST matches in NCBI are shown in brackets): Sclerotiniaceae (several species, blast hits including both *Botrytis cinerea* and *S. sclerotiorum*), *Blumeria* (*B. graminis), Monographella (Microdochium nivale), Venturia* (several species), *Pyrenophora* (*P. teres* and *P. bromii*), *Phoma* (several species), *Claviceps* (*C. purpurea*), *Phaeosphaeria* (several species), Mycosphaerellaceae (mainly *Ramularia* infecting trees such as *Malus* and *Sambucus*), and *Tilletia* (*T. walkeri* and *T. caries*).

Sclerotiniaceae (13700 reads), Mycosphaerellaceae (10060 reads), *Blumeria* (2583 reads), and *Microdochium* (1681 reads) were found in relatively high abundance, whereas reads from the other genera were found in much lower abundance. *Blumeria* reads could be split into different ‘formae specialis’ (*B. graminis* f.sp. *hordei*, *B. graminis* f.sp. *tritici*), however, the vast majority of reads belonged to *B. graminis* f.sp. *tritici* (data not shown). There was a remarkably high abundance of the class Exobasidiomycetes in the Rothamsted field samples from late spring 2012 (**Figure 1**), a closer inspection by BLAST matches of representative reads revealed that they belonged to the genus *Entyloma*, a genus consisting of plant pathogenic smut fungi. The genus *Leptosphaeria*, containing the oilseed rape pathogen *L. maculans* was found in low amounts primarily in samples from autumn, and a qPCR confirmed the presence of this pathogen (**Supplementary Table S1**).

**FIGURE 5:**
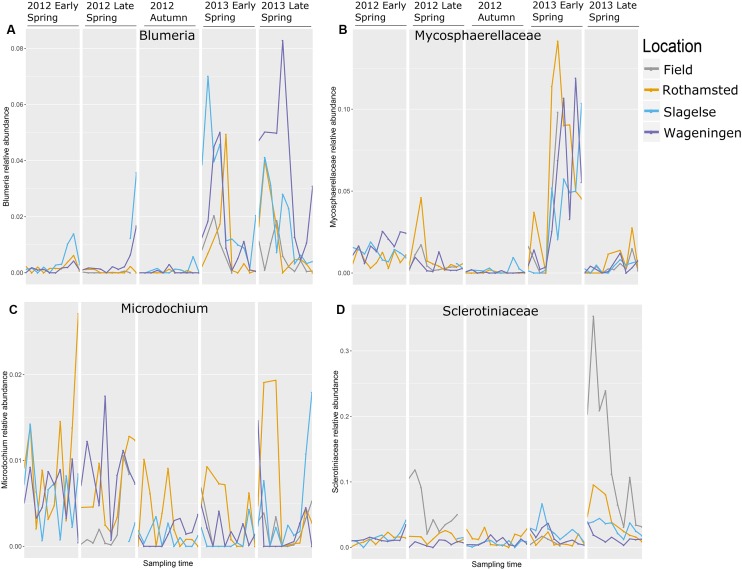
The most abundant genera with mainly plant pathogenic during five periods in spring 2012 and 2013 and autumn 2012. Note that the graph does not represent a continuous sampling but five temporally separated periods. **(A)** *Blumeria*; **(B)** Mycosphaerellaceae; **(C)** *Monographella* (*Microdochium*); **(D)** Sclerotiniaceae.

The relative abundance of taxa containing plant pathogens varied greatly over the sampling periods in 2012 and 2013 as exemplified in **Figure 5** by the four most abundant taxa. Read distributions were strikingly similar at the three locations for many genera including *Blumeria* and Mycosphaerellaceae which were both present in higher quantities in spring 2013 compared to spring 2012 and were almost absent in autumn 2012 (**Figures 5A,B**). In contrast, *Monographella* was more evenly distributed throughout the different periods (**Figure 5C**), although significant day-to-day variation could be observed. Sclerotiniaceae was highly abundant in samples from above the oilseed rape fields in the late spring 2013 (**Figure 5D**), which was confirmed by qPCR analysis (**Supplementary Table S1**).

**FIGURE 6:**
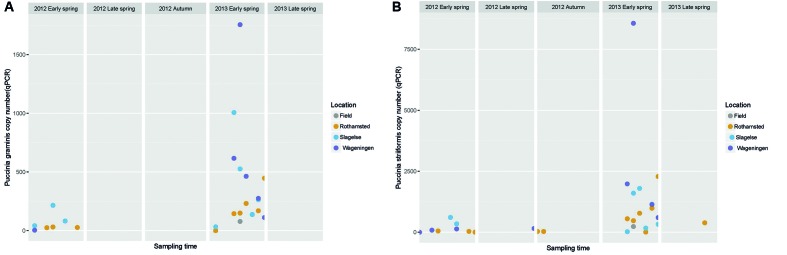
Graph showing the abundance of *Puccinia graminis* and *P. striiformis* gene copy numbers quantified by qPCR in Rothamsted, Slagelse, and Wageningen during five periods in spring 2012 and 2013 and autumn 2012. Note that the graph does not represent a continuous sampling but five temporally separated periods. **(A)** *P. graminis*; **(B)** *P. striiformis*.

The amount of the plant pathogens *P. striiformis*, *P. graminis* and *P. triticina* in the samples was quantified using qPCR. This revealed peaks of *P. striiformis* and *P. graminis* in the early spring of 2013 but also a smaller peak in early spring of 2012 (**Figure 6**).

## Discussion

This is one of the first studies to examine fungal diversity in air samples using metabarcoding. On the basis of relative quantities of reads we studied the composition of fungal taxa in air sampled at three locations representing different Northwestern European regions. To dilute effects from single close-by fields, spore traps were placed on top of tall buildings in urban surroundings. For comparison, we also included a few samples taken from the air just above oilseed rape fields in Rothamsted, UK. The same air sampler type was used in all locations (a Hirst-type, Burkard 7-day recording volumetric spore trap), as this is known to have good reliability, operates over a wide range of typical wind speeds and has excellent collection efficiency of particles over a broad size range (>50% collection efficiency for particles with aerodynamic diameter over 2.2 μm), therefore a slight under-sampling of the very smallest fungal spores was likely (West and Kimber, 2015). The study clearly demonstrated that the composition of fungal communities in air was similar over a large geographical area (Northwestern Europe) in a region with similar climates. As expected, season had a marked influence on fungal composition probably reflecting the different climatic conditions, phenological patterns, and different life cycles of the fungal taxa and the varying availability of fungal growth substrates during the year.

Although sampling sites were separated by up to 900 km (between Slagelse and Rothamsted), location did not have significant effects on observed species richness between the three locations. Based on variance partitioning on community distances by the *adonis* test, location explained 8.0% of the variance in the dataset. Likewise, a PCoA plot did not show any distinctive clustering based on sampling site again confirming that location within this climatic region is not an important driver of the diversity of airborne fungal spores. This observation is supported by Bowers et al. (2013), who only found differences in the relative abundance of bacterial domains, but not in the relative abundance of fungi at two different locations in Colorado, United States. The three locations used in this study are all in the Northwestern part of Europe with approximately identical climates (mild winters, cool summers with an average yearly rainfall between 750 and 850 mm), and with similar plant and crop covers. In addition, rooftop sample locations at heights around 10–15 m are expected to sample air well-mixed by the turbulent boundary layer and representing particles released from many sources over a relatively large local area compared to ground locations, which bias sampling toward close local sources rather than the background air stream (Lacey and West, 2006). This may explain the little variation observed between the three sampling sites, as the majority of airborne spores originate from local plant and soil sources and only a minority originates from distant sources transported by strong winds (Behzad et al., 2015). The species present in the air spora at the three sites appears to be similar at each sample period when generally mobile patterns of wind circulation (direction) in Northwestern Europe delivered similar weather differing only in timing of passing weather systems. Differences between sites could be more apparent on certain individual days, because as weather systems pass over NW Europe, the wind direction either side of the weather front is different (Schmale and Ross, 2015) and also due to local active dispersal of spores after a rain front has delivered rain compared to dry conditions locally ahead of the front. Finally, DNA markers with a finer resolution might have revealed subtle differences in the read abundances between closely related strains that were not discernible using the ITS1 region as a marker.

There was generally a lower amount of PCR product from samples taken in autumn than from samples taken in the spring (data not shown), which may be caused by lower amounts of airborne fungal spores in autumn, although other explanations may exist such as differences in PCR inhibition, differences in the lysis of spores, or differences in trapping efficiency in samples between the seasons. Despite this, species richness was highest in autumn compared to early and late spring. This could be caused by the higher amounts of decaying plant material after the growth season, a more diverse plant cover after harvest of monoculture crops, or by the abundance of sporulating mushrooms in the autumn. The higher proportion of Basidiomycota during autumn was mainly caused by Agaricomycetes including many mushroom-forming fungi that are dominant in autumn in the Northwestern European area. This last observation is supported by Fröhlich-Nowoisky et al. (2009) who also found a higher relative abundance of Agaricomycetes during this period. The finding that species richness is higher in the autumn is partly supported by Yamamoto et al. (2012) who analyzed fungal communities during a year in air samples taken from a rooftop of a five-story building in New Haven, CT, United States and by Fröhlich-Nowoisky et al. (2009) who analyzed air samples from a rooftop of a three-story building in Mainz, Germany. Although not significant, Fröhlich-Nowoisky et al. (2009) did observe slightly higher richness during autumn, whereas Yamamoto et al. (2012) found higher concentrations of fungi (although not higher richness), also during autumn. These different observations may be caused by local differences such as variations in plant-covered areas and diversity of plant species in surroundings.

The species richness in field samples was low compared to rooftop samples, probably caused by dominance of spores from a few fungal species coming from the crop just below the spore sampler. Lower diversity is expected at ground level than at rooftop height because the air is less-well mixed and the sample is heavily weighted toward sources of spores close to the sampler because less dilution will have occurred into the atmosphere. This suggests that the rooftop site is actually a better location for assessing fungal content in air representative of the region and to detect more exotic airborne particles than at ground level. The rooftop samples represent smoothed samples of mixed air comprising spore releases from many different microclimates and habitats within the region.

In field samples, Exobasidiomycetes were highly dominant at single days or at short intervals of days in late spring 2012. Sequence comparisons showed that reads from Exobasidiomycetes belonged to *Entyloma*, a genus in which the plant pathogenic smuts belong. We were not able to identify reads to species level, however, there were fields of grass nearby and long uncut grass margins around the field that had not been fungicide treated, these may have been infected with smuts providing a link to observations in the air sample data. The Sclerotiniaceae dominated a few samples during late spring 2013. This indicates massive spore release from the crop just below the sampler, which was oilseed rape that had been artificially inoculated with *S. sclerotiorum*. The oilseed rape crop was also affected by *L. maculans* which produce airborne spores in the autumn and winter. This corresponds well with the presence of reads of this species in the autumn samples, as well as our qPCR data.

Based on variance partitioning on community distances, season explained 24.6% of the variance. A PCoA plot illustrated clustering of samples according to both year and season. Early spring and late spring samples showed some clustering but not as clear as between spring and autumn or between the two years. Early spring was sampled in the end of April (2012) or beginning of May (2013) and late spring was sampled at the beginning of June (both years) meaning that the two sampling periods were only separated by approximately 1 month, which may explain the similarity between fungal communities in the two sampling periods. The two years 2012 and 2013 were clearly separated. This may be explained by differences in the weather conditions during the different periods or by differences in plant cover between the 2 years. Generally spring 2012 was wet, autumn 2012 was slightly wetter than normal, and spring 2013 was relatively dry and 2013 was warmer than 2012 during sampling periods (**Supplementary Table S2**).

Different fungal genera containing important plant pathogens showed different patterns of relative read abundances during the season, but were strikingly similar across the three locations and in many cases peaking on approximately the same days as shown for *Blumeria*, Mycosphaerellaceae, *Monographella* and Sclerotiniaceae. *Blumeria* causes powdery mildew in grasses including cereals and is an obligate parasite depending on living host tissue. It overwinters as chasmothecia in plant debris or as mycelium and conidia in living plant tissue. In early spring airborne ascospores from chasmothecia is the primary inoculum but also conidia from overwintering mycelium is released. *Blumeria* reads showed the highest peaks in 2013 in all three locations with an initial peak in early spring and a later peak in late spring. Interestingly, many of the peaks coincided on the same day at the three locations. However, our data set is too limited to predict which factors have provoked the massive spore releases. The wet spring of 2012 was not conducive to a strong epidemic, while relatively dry and warmer conditions in 2013 (but still with some rain occasionally) probably facilitated better sporulation and dispersal events. *Blumeria* was almost absent from autumn samples, which is explained by the fact that very little green plant substrate is available at that time. *Blumeria* peaks from samples taken from rooftops coincided with peaks from air samples taken from above crops, indicating dispersal of *Blumeria* spores from the surrounding rural areas at the regional scale.

Mycosphaerellaceae peaked in early spring 2013 at all three locations. The majority of reads assigned to this family were identified as belonging to *Ramularia* and some could be even further identified to species such as *R. vizellae, R. unterseheri*, and *R. endophylla.* These are species that infect trees such as alder, oak and beech, which are grown in many urban areas. The peak in early spring 2013 was probably originating from an early onset of *Ramularia* in one or more tree species in the urban surroundings caused by the warm and dry conditions in spring 2013. Infection by *Ramularia* is initiated by air-borne ascospores and splash-dispersed conidia produced on leaf residues from the previous season. Surprisingly, we did not detect any *Zymoseptoria tritici* reads in our dataset. *Z. tritici* causes septoria leaf blotch, one of the most important diseases in wheat. However, other studies also found very low levels of this pathogen in air samples taken during early spring with airborne ascospore counts peaking later in the season (July/August) (Fraaije et al., 2005; Duvivier et al., 2013).

The high peak of Sclerotiniaceae in the Rothamsted field samples coincided with data from the Rothamsted rooftop samples in late spring 2013. This field was artificially inoculated with *S. sclerotiorum* for a different experiment. It is interesting that the rooftop site had a similar pattern of *S. sclerotiorum* spores even though it was 1.3 km away and with the wind often not blowing from the field site. This suggests that the rooftop site was sampling spores from natural sources in the region which responded to the same weather events to release ascospores. *Monographella* (identified by BLAST in NCBI to *Microdochium nivale*) did not show a marked seasonal variation, although there was a high day-to-day variation and the abundance was higher in spring 2012. This could reflect the fact that *M. nivale* conidia or ascospores can be produced from plant debris all year round and also from, e.g., susceptible weed species (Landschoot et al., 2011) whereas other fungi may have narrower host ranges or, such as *Blumeria*, require a living host for spore production. Furthermore, *M. nivale* prefers cool and humid conditions, conditions that were present in spring 2012. The presence of *Puccinia* sp. in the samples especially in early spring where *P. striiformis* was detected in the beginning of May, 2013 at all three locations and at exactly the same period (May 7 to 12), demonstrating strikingly simultaneous peaks of inoculum of some pathogens at the three locations, as was also observed for *Blumeria* and *Mycosphaerella*. Generally, our results show that air samples may be an important source for monitoring movements of *Puccinia* spores on a regional scale.

Monitoring air-borne inoculum of plant pathogens is relevant for developing decision support systems for disease management and also for understanding fundamental questions in epidemiology of plant pathogens, even more so in view of climate change which is predicted to increase severity of many plant diseases. Until recently, monitoring was done by microscopy and in some cases by culturing of the organisms, or more recently, by PCR-based methods able to monitor individual pathogens of interest at a high sensitivity and specificity. Using next generation sequencing based metabarcoding, it is now possible to analyze the composition of fungal communities in air samples at a very high resolution and in practice all pathogens in one analysis, including unculturable organisms. This opens new opportunities to study the temporal and spatial dynamics of spore concentrations, the factors that shape these communities, and finally how these concentrations influence disease development of crops. However, there are still many challenges for the full exploitation of metabarcoding in epidemiological studies of plant pathogens: (i) as most plant pathogens have a narrow host range it is necessary to identify reads at species level or even below this. This represents a challenge, as only a fraction of reads can be assigned to species level when using the ITS region as the barcode; (ii) as disease may be initiated from small numbers of spores, a very high sensitivity is required in a high background of other material. It has not yet been fully investigated whether metabarcoding has sufficient sensitivity for detection of primary inoculum concentrations; (iii) PCR procedures may introduce biases in read abundance as shown in the present experiment, in which no *Puccinia* was detected using metabarcoding, although spores of *Puccinia* were present, as evidenced by qPCR; (iv) method and location of sampling may have impact on data. Generally, qPCR approaches have shown very high sensitivity and will be the preferred method for detection of single species of pathogens because of both sensitivity and specificity whereas metabarcoding has higher potential for a systems-based understanding of microbial diversity in air.

In this study we have found that the relative abundance of fungal spores does not vary much over relatively large areas with similar climates, and that some species of pathogens peak at the same days even at long distances apart. The explanation for this could be due to the sampling locations having similar climates and land-use surrounding them. However, further work is needed as our data were too limited to link weather observations and land-use for the three locations to metabarcoding data.

## Author Contributions

MN, JW, CS, and AJ designed the experiment. MN, JW, GC, CS, and AJ sampled and performed the laboratory work. MN, JW, RS, CS, and AJ analyzed experimental data. MN, JW, and AJ prepared the manuscript. All authors approved the manuscript for submission.

## Conflict of Interest Statement

The authors declare that the research was conducted in the absence of any commercial or financial relationships that could be construed as a potential conflict of interest.

## Supplementary material

FIGURE S1 Species accumulation curve showing the number of detected OTUs as a function of number of samples.

TABLE S1 Operational taxonomic unit (OTU) table including taxonomic classification, sequencing run, barcode used, location, date of sampling and relative abundance of reads. Results from qPCR assays of *Puccinia graminis, P. striiformis, P. triticina, Leptosphaeria maculans, Sclerotinia sclerotiorum*, and *Microdochium nivale* are given as gene copy numbers or DNA mass (ng); nd, not detected.

TABLE S2 Weather data on the days of sampling on the three sampling locations, Rothamsted, UK, Slagelse, DK, and Wageningen, NL. Daily precipitation and mean temperatures are given.

TABLE S3 Observed species at the different locations. Significance of differences was tested using the two-sided Student’s two-sample *t*-test.

TABLE S4 Primer and probes: (a) developed probe sequence (Kim and Knudsen, 2011) was modified for the reporter dye, (b–d) developed probe sequences (Barnes and Szabo, 2007) were modified with a ZEN component and quenching IABkFQ dye, (e) primers and probe (Waalwijk et al., 2004), (f) primers and probe were developed by PRI (Wageningen, NL). All primers and probes were supplied by IDT (United States).
