# Supplementary figures and images for: Fungal Communities Including Plant Pathogens in Near Surface Air Are Similar across Northwestern Europe
Source: Front Microbiol. 2017 Sep 8;8:1729. doi: 10.3389/fmicb.2017.01729 (PMC5596660; doi:10.3389/fmicb.2017.01729)

Number of fungal OTUs

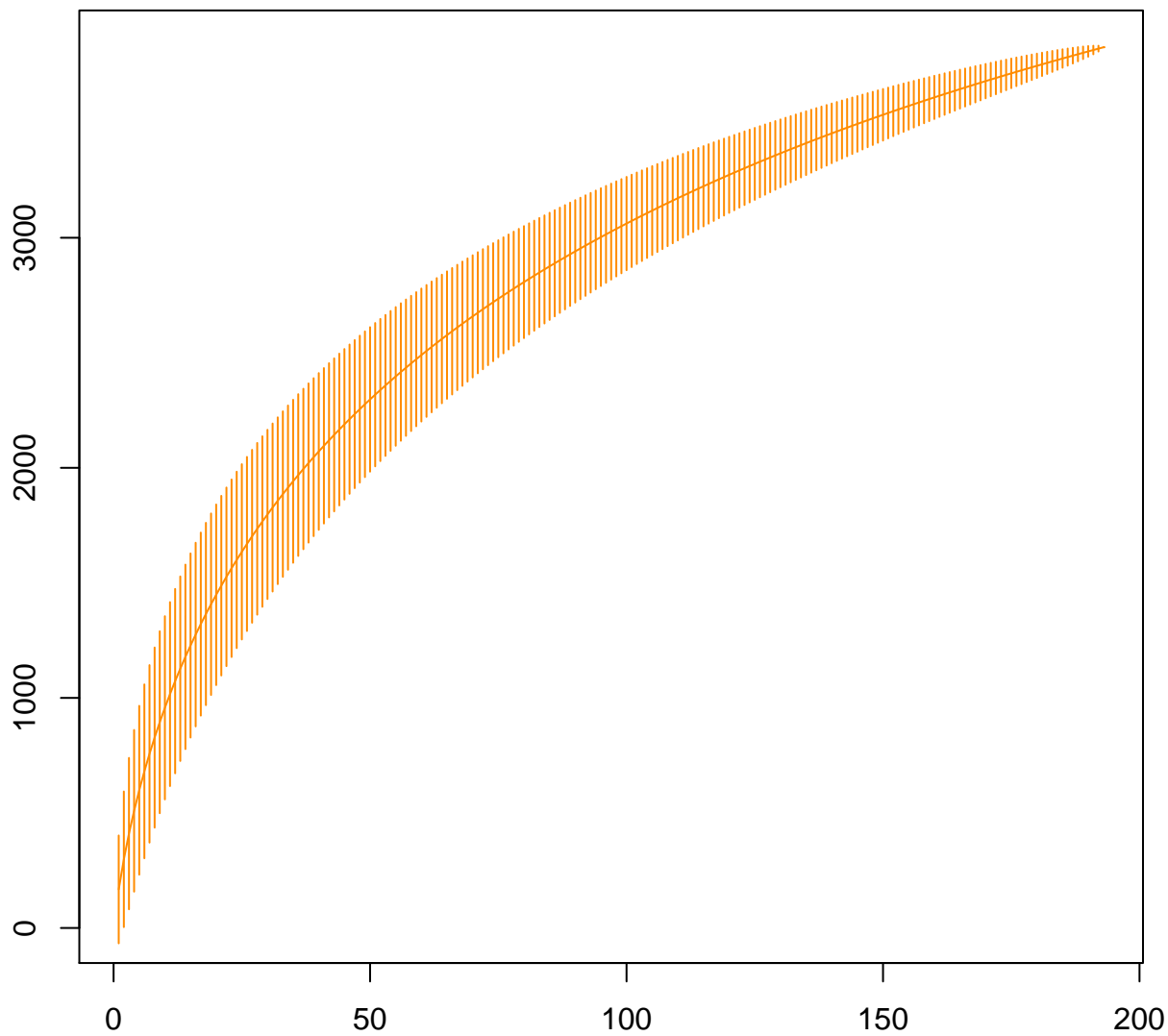

Supplement: FIGURE S1 — Species accumulation curve showing the number of detected OTUs as a function of number of samples. [file Image_1.PDF]
