# Supplementary material for: Fungal Communities Including Plant Pathogens in Near Surface Air Are Similar across Northwestern Europe
Source: Front Microbiol. 2017 Sep 8;8:1729. doi: 10.3389/fmicb.2017.01729 (PMC5596660; doi:10.3389/fmicb.2017.01729)
Supplement: TABLE S4 — Primer and probes: (a) developed probe sequence (Kim and Knudsen, 2011) was modified for the reporter dye, (b–d) developed probe sequences (Barnes and Szabo, 2007) were modified with a ZEN component and quenching IABkFQ dye, (e) primers and probe (Waalwijk et al., 2004), (f) primers and probe were developed by PRI (Wageningen, NL). All primers and probes were supplied by IDT (United States). [file Table_4.DOCX]

|  | **Target** | **Target position** | **Forward primer** sequence (5’-3’) | **Reverse Primer** sequence (5’-3’) | | **TaqMan Probe** sequence (5’-3’) | |  | |  | |  |
| --- | --- | --- | --- | --- | --- | --- | --- | --- | --- | --- | --- | --- |
| ***a*** | *Sclerotinia sclerotiorum* | Calmodulin | CCCAGTTCGACTCTCCTCTTTTAT | AACTCAGACTCGGAAGGGTTTTG | | 6-FAM AGACATCTTGACCGACACCGCCCC BHQ1 | |  | |  | |  |
| ***b*** | *Puccinia striiformis* | ITS1 | TGAACCTGCAGAAGGATCATTA | TGAGAGCCTAGAGATCCATTGTTA | | 6-FAM TAAGACTTG/ZEN/GTTGCATGATTTGAAAGAATCATT IABkFQ | |  | |  | |  |
| ***c*** | *Puccinia graminis* | ITS1 | TGAACCTGCAGAAGGATCATTA | TGAGAGCCTAGAGATCCATTGTTA | | 6-FAM TTGTGGCTC/ZEN/GACTCTCTTATAAACCAAACC IABkFQ | |  | |  | |  |
| ***d*** | *Puccinia triticina* | ITS1 | TGAACCTGCAGAAGGATCATTA | TGAGAGCCTAGAGATCCATTGTTA | | 6-FAM TGAAAGAAT/ZEN/CATTGTGATTAAGTATACGTGGCATTCT IABkFQ | |  | |  | |  |
| ***e*** | *Microdochium nivale* | SCAR | CGCCAAGGACTCCTCCAGTAG | GCCGACGAATGGATATTAAGAACT | | 6-FAM TCCCGCCTTCACGGTGGAAAGC BHQ1 | |  | |  | |  |
| ***f*** | *Leptosphaeria maculans* | Actin | cgcgcaggaaaacagattttt | gaagctggaattgagttagcatgtac | | 6-FAM cgtgcttctgccggctctagcg BHQ1 | |  | |  | |  |
|  |  |  |  | |  | |  | |  | |  | |
